# Self-management Practices among U.S. Chinese older adults with Major Depressive Disorder

**DOI:** 10.1093/geroni/igaf122.2714

**Published:** 2025-12-31

**Authors:** Ying-Yu Chao, Jin Young Seo, An-Yun Yeh, Weiming Wang, Yu-Ping Chang

**Affiliations:** Rutgers University, Newark, New Jersey, United States; Hunter College, New York, New York, United States; The College of Staten Island, CUNY, Staten Island, New York, United States; New York University Rory Meyers College of Nursing, New York, New York, United States; University at Buffalo, Buffalo, New York, United States

## Abstract

**Purpose:**

Depression is common in older immigrants due to low levels of acculturation, language barriers, and social isolation. In Chinese communities, the cultural stigma surrounding mental health further exacerbates these challenges. This study aimed to enhance the understanding of self-management strategies and barriers among U.S. Chinese older adults living with Major Depressive Disorder (MDD).

**Methods:**

A qualitative study design was employed. Participants were recruited from a psychiatric clinic in New York City. A demographic survey was administered to collect participants’ characteristics. Semi-structured individual interviews were conducted to explore their self-management strategies for depressive symptoms. Data were analyzed using descriptive statistics and thematic analysis.

**Results:**

Seventeen patients with MDD were recruited. The average age was 60.53 (SD: 6.47; range: 51-75) years, 52.9% were male, and 70.6% were married. Self-management strategies included seeking help and adhering to treatment, finding comfort in supportive relationships and social engagement, engaging in enjoyable activities, and practicing acceptance and letting go. Challenges to self-management included failing to recognize underlying emotional problems, fighting cultural stigmas and misconceptions, encountering help-seeking barriers as immigrants, and ongoing struggle with medication reliance and concerns about side effects.

**Conclusions/Implications:**

Participants developed individualized strategies to manage depressive symptoms. Health care providers should assess older adults’ perceptions of depression, understand the challenges they face in self-management, and provide tailored interventions for those struggling to manage their symptoms. Moreover, efforts to deliver culturally sensitive, collaborative care should focus on enhancing mental health literacy, increasing public awareness, and implementing anti-stigma interventions for this population.

